# Supplementary material for: Plasma metabolomic profiling in patients with rheumatoid arthritis identifies biochemical features predictive of quantitative disease activity
Source: Arthritis Res Ther. 2021 Jun 8;23:164. doi: 10.1186/s13075-021-02537-4 (PMC8185925; doi:10.1186/s13075-021-02537-4)
Supplement: Supplementary file 1 — Additional file 1: Supplementary Figure 1. Histogram of DAS28-CRPs corresponding to the 128 total samples of the discovery cohort. [file 13075_2021_2537_MOESM1_ESM.pdf]

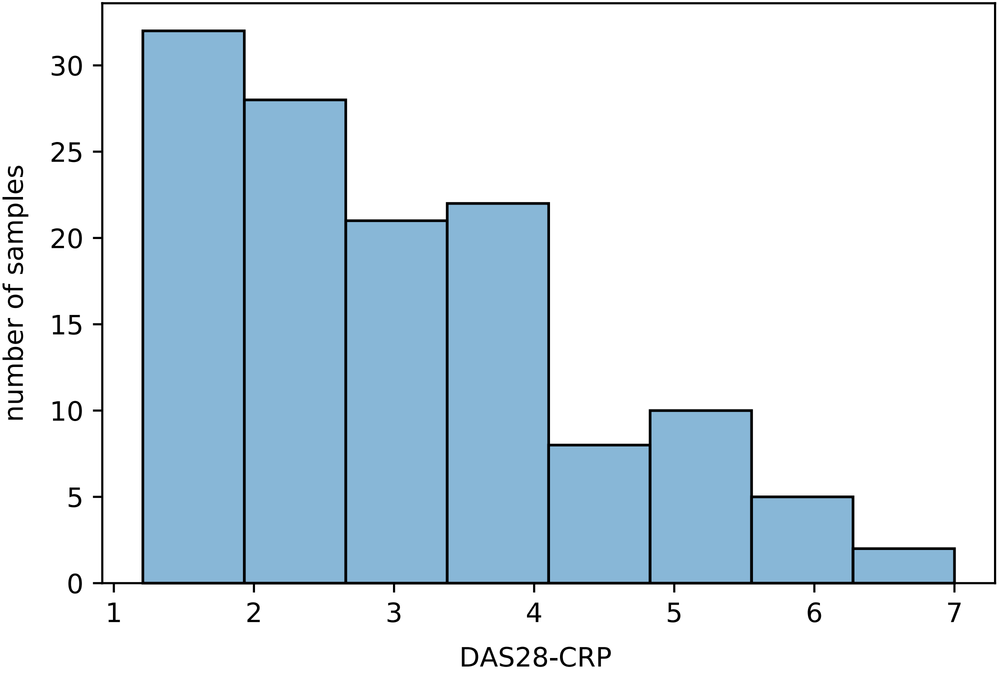

Supplementary Figure 1. Histogram of DAS28-CRPs corresponding to the 128 samples of the discovery cohort.
